# Melanoma vaccines: current R&D landscape, translational hurdles, and future outlook—a perspective drawn from 442 clinical trials

**DOI:** 10.3389/fimmu.2025.1698503

**Published:** 2026-01-19

**Authors:** Canni Gao, Xinxin Duan, Leixuan Peng, Yixin Zhao, Pan Li, Kuanhou Mou

**Affiliations:** 1 Department of Dermatology, The First Affiliated Hospital of Xi’an Jiaotong University, Xi’an, China; 2 Center for Translational Medicine, The First Affiliated Hospital of Xi’an Jiaotong University, Xi’an, China

**Keywords:** melanoma, cancer vaccine, clinical trials, combination therapy, immunotherapy

## Abstract

**Background:**

Melanoma, a highly malignant skin tumor with high metastatic propensity and poor survival in advanced stages, poses a major global public health challenge, as conventional treatments have notable limitations. Tumor immunotherapy, particularly cancer vaccines, has emerged as a promising approach by activating/regulating immune mechanisms to target cancer cells.

**Methods:**

This study systematically searched the Trialtrove database for interventional clinical trials of melanoma and cancer vaccines up to August 5, 2025. After screening via inclusion/exclusion criteria, 442 trials were analyzed, adhering to PRISMA guidelines with independent dual review for data reliability.

**Results:**

Trials were geographically concentrated in developed regions (69% in the US), with minimal participation from Asia, Africa, and Latin America. A “translational funnel effect” was observed: Phase I/I-II trials accounted for 63.6%, while Phase III trials only 6.1%, with a 22.9% termination rate. Peptide/recombinant protein vaccines (186 trials) and cellular vaccines (151 trials) were mainstream, with nucleic acid vaccines (58 trials) as a promising emerging platform. Combination therapy (227 trials, >50%), especially with immune checkpoint inhibitors (ICIs), dominated; adjuvants (e.g., IL-2, GM-CSF agonists) enhanced efficacy. Most trials focused on Stage III/IV patients (91.1%): key trials showed mRNA-4157 + pembrolizumab reduced recurrence/death risk by 49% in resected melanoma, and herpes simplex virus RP1 + nivolumab achieved 58.3% objective response rate (ORR) in ICI-resistant patients. Primary endpoints favored safety/immunogenicity (215/142 trials), with overall survival (OS, 33 trials) rarely used; academic institutions led funding (52.3%).

**Conclusions:**

Melanoma vaccines, especially in combination with ICIs and via personalized platforms, have significant potential. However, challenges include tumor heterogeneity, immunosuppressive tumor microenvironment (TME), inefficient delivery, geographical R&D imbalance, and low Phase III conversion. Interdisciplinary collaboration, international multicenter trials, optimized clinical design (e.g., early-stage patient enrollment), and policy support are needed to advance their clinical translation.

## 1 Introduction

Melanoma is the most aggressive subtype of skin cancer, characterized by strong invasiveness and metastatic potential. It can spread to distant organs such as the lungs, liver, and brain via lymphatic and hematogenous pathways even at an early stage, resulting in an extremely poor prognosis for patients (1). At the molecular level, melanoma cells can enhance their invasive capacity by activating signaling pathways such as AXL; among patient populations, elderly male patients are more prone to treatment resistance and metastasis due to elevated secretion of bone morphogenetic protein 2 (BMP2) in the tumor microenvironment (2). In terms of treatment, while early-stage melanoma can be cured in some patients through surgical resection, the 5-year survival rate for advanced-stage patients is only 6% (3). Globally, the incidence of melanoma continues to rise, with particularly significant increases observed in Europe and North America (4). Its high mortality rate is closely associated with treatment resistance: conventional surgery, radiotherapy, and chemotherapy have limited efficacy in advanced-stage patients; even in immunotherapy, the objective response rate (ORR) remains relatively low, with only approximately 10%-15% of patients achieving partial response (PR) (5). Therefore, the development of novel therapeutic strategies has become an urgent need in the current field of melanoma research.

As an active immunotherapy, the core objective of cancer vaccines is to stimulate or reconstitute the body’s anti-tumor immune responses, enabling the specific recognition and long-lasting elimination of tumor cells (6). Their mechanism of action is primarily achieved through the following pathways: by delivering tumor-specific antigens (TSAs) or tumor-associated antigens (TAAs), and relying on adjuvant systems and delivery technologies to activate antigen-presenting cells (APCs)—with dendritic cells (DCs) as the core—subsequently initiating cytotoxic T lymphocyte (CTL) responses and ultimately inducing tumor killing (7). Based on differences in technical platforms, cancer vaccines are mainly categorized into four types, with the characteristics and modes of action of each type outlined below: (1)Protein Vaccines: This category includes synthetic peptide vaccines and recombinant protein vaccines. Protein vaccines obtain tumor-associated antigens (TAAs)/tumor-specific antigens (TSAs) via chemical synthesis or recombinant expression technologies, and often rely on adjuvants to enhance their immunogenicity. The antigens are directly delivered to the immune system, inducing specific antibody and T-cell responses.(2)Nucleic Acid Vaccines: This category encompasses mRNA vaccines and DNA vaccines. By delivering mRNA or DNA encoding tumor antigens, nucleic acid vaccines utilize host cells to express the target antigen while triggering both humoral and cellular immune responses. They possess the advantages of flexible development and convenient production.(3)Cellular Vaccines: Examples include dendritic cell (DC) vaccines and whole-tumor cell vaccines. This type of vaccine uses exogenously antigen-loaded DCs (e.g., Provenge^®^, the approved prostate cancer therapeutic vaccine) or processed tumor cells as carriers. Leveraging their robust antigen-presenting capacity, they effectively activate antigen-specific T-cell immunity.(4)Viral Vector Vaccines: Replication-defective viruses (e.g., adenoviruses, poxviruses) are typically used as vectors (such as the Ad5-vectored COVID-19 vaccine). These vectors deliver genes encoding tumor antigens into host cells, mimic the natural infection process, enable efficient antigen expression, and elicit a strong T-cell-dominant immune response (8, 9). Additionally, other emerging platforms are also under active exploration: Bacterial vector vaccines: Employ genetically engineered attenuated live bacteria (e.g., Listeria monocytogenes) as carriers. By integrating tumor antigen genes into the bacterial genome, the bacteria express target antigens *in vivo*, triggering a strong immune response dominated by cellular immunity (10).Exosome-based vaccines: Utilize naturally secreted nanoscale vesicles from cells as delivery systems. Through engineering modification (surface display/internal encapsulation of antigens), they leverage high biocompatibility and targeting ability to synergistically activate both humoral and cellular immune responses (11).

Currently, research on cancer vaccines focuses primarily on three core directions: technological innovation, optimization of combination therapy strategies, and development of personalized vaccines. At the level of technological innovation, vaccine design has shifted from a single-antigen paradigm to a precision-oriented, multi-targeted model. On the one hand, novel delivery technologies have significantly improved immune efficacy: mRNA vaccines (with the advantage of rapid customization, e.g., mRNA-4157, which can encode 34 neoantigens), the high-efficiency delivery properties of DNA nanostructures, and the antigen transfer mechanism of biomimetic nanovaccines have greatly enhanced immunogenicity and activation efficiency (12, 13). On the other hand, adjuvant systems have overcome application bottlenecks through multi-pathway synergy (e.g., the SABER adjuvant simultaneously targeting the endoplasmic reticulum (ER) and STING pathways) and innovations in industrialization technologies. For instance, the EVX-01 personalized tumor vaccine screens tumor neoantigens via an AI platform and is formulated with the novel adjuvant CAF^®^09b, with a production cycle of only 48–55 days—laying a foundation for the large-scale application of personalized vaccines (14). In terms of combination therapy strategies, the sequential/simultaneous administration of vaccines with immune checkpoint inhibitors (ICIs), the immune-sensitizing effect of radiotherapy and chemotherapy, and pathway synergy with targeted drugs can reverse T cell exhaustion through functional complementarity, while enhancing immune memory and long-term anti-tumor effects. In the phase Ib clinical trial (NCT02897765) conducted by Patrick A. Ott et al., efficacy data for the combination of NEO-PV-01 and the anti-PD-1 agent nivolumab in patients with advanced melanoma demonstrated significant tumor regression activity, achieving an objective response rate (ORR) of 59%. Regarding progression-free survival (PFS), the median PFS of melanoma patients who received the vaccine was 23.5 months, which was significantly longer than historical data for anti-PD-1 monotherapy (15). At the level of personalized vaccine development, multiple technical routes have demonstrated unique value: The biomineralized tumor nanovaccine (NV) developed by the Sijia Zhang team integrates the model antigen ovalbumin (OVA), CpG adjuvant, and manganese nanoparticles in a one-stop manner, achieving efficient delivery and immune activation. When combined with anti-PD-L1 antibodies, this vaccine can prevent tumorigenesis, inhibit metastasis, and induce long-lasting immune memory. Furthermore, the personalized nanovaccine (PNV) developed by the same team—constructed based on tumor tissue fluid from surgically resected tumors—more significantly inhibits postoperative recurrence, highlighting the design advantage of personalized antigen sources (16). Additionally, the hybrid M13 phage-based personalized vaccine (HMP@Ag) developed by the Xue Dong team delivers tumor-specific antigens via phage vectors, activates antigen-presenting cells (APCs), and enhances immune responses through the TLR9 signaling pathway. This vaccine exhibits both preventive and therapeutic effects in multiple tumor models; when combined with immune checkpoint blockade (ICB), it can slow tumor growth, inhibit postoperative recurrence, and induce long-lasting immune memory, demonstrating broad clinical potential (17).

Despite these advancements, the clinical translation of cancer vaccines still faces key challenges: incomplete antigen coverage caused by tumor heterogeneity; the antagonistic effects of regulatory T cells (Tregs) and myeloid-derived suppressor cells (MDSCs) in the immunosuppressive tumor microenvironment; low targeting efficiency of delivery systems (e.g., the lymph node targeting rate of nanocarriers is < 10%); and high cost and limited accessibility of personalized vaccines (18). Against this backdrop, this study will analyze cancer vaccine clinical trials conducted in recent years, with a focus on the efficacy and limitations of different technical routes. It aims to provide a theoretical basis and data support for the optimized design of melanoma vaccines and the formulation of combination therapy strategies, thereby facilitating their clinical translation and application.

## 2 Method

### 2.1 Data sources and search strategy

This study conducted a systematic search of the Trialtrove database (Citeline Group), an authoritative global repository of clinical trials, to identify interventional trials investigating cancer vaccines for melanoma. The search was executed on August 5, 2025, with a comprehensive strategy designed to encompass all relevant records registered up to that date. The specific parameters were as follows: disease, melanoma; intervention, cancer vaccine; study type, interventional trials. No date restrictions were applied.

### 2.2 Trial screening and data extraction

The initial search yielded a broad set of records. The screening process comprised two stages: (1) Title/Abstract Screening: all records were screened to exclude observational studies and trials in which the primary intervention was not a cancer vaccine; (2) Full-Text Assessment: the remaining trials underwent a full-text review to confirm their eligibility based on predefined criteria. The inclusion criteria were interventional clinical trials focusing on therapeutic or prophylactic cancer vaccines for melanoma. The exclusion criteria were: (i) observational studies; (ii) trials where the vaccine was not the primary intervention; (iii) trials for non-melanoma cancers; and (iv) duplicate records. A total of 442 trials were finally included. The screening process adhered to the PRISMA (Preferred Reporting Items for Systematic Reviews and Meta-Analyses) guidelines (19). Data from the included trials were systematically extracted into a standardized spreadsheet, including: trial identification number (NCT ID) and title; start year and completion status; trial phase; recruitment status; geographic locations of trial sites; planned/enrolled sample size; melanoma stage of participants; vaccine platform/type; mechanism of action (MoA) category; combination therapies; primary and secondary endpoints; sponsor type; and key agents.

### 2.3 Categorization of vaccine platforms

Vaccines were categorized into four main platforms based on their core technological composition: (1)Peptide/Protein Vaccines, which include synthetic short or long peptides and recombinant proteins encoding tumor-associated antigens(TAAs)or tumor-specific antigens (TSAs), typically administered with an adjuvant; (2) Cellular Vaccines, comprising whole-cell–based approaches such as ex vivo antigen-loaded dendritic cell (DC) vaccines or autologous/allogeneic whole tumor cell vaccines; (3) Nucleic Acid Vaccines, including DNA and mRNA platforms that deliver genetic material encoding tumor antigens for in vivo expression by host cells; (4) Viral Vector Vaccines, which use replication-competent or incompetent viruses (e.g., adenovirus, poxvirus) to deliver antigen genes.

### 2.4 Categorization of mechanism of action (MoA)

The primary immunological mechanism of the vaccine intervention was categorized based on the stated target or function described in the trial protocols: (1) Immune Activation and Stimulation: Strategies aimed at directly enhancing effector functions of the immune system, including the use of T-cell stimulants, immunostimulants, GM-CSF agonists, lymphocyte stimulants, and Toll-like receptor agonists; (2)Immune Checkpoint Targeting: Approaches designed to block inhibitory signals on T cells to reverse immune tolerance in the tumor microenvironment, primarily involving PD-1/PD-L1 antagonists and CTLA-4 antagonists; (3)Cytokine and Receptor Targeting: Interventions that modulate immune responses by regulating key cytokine pathways, such as interleukin-2 agonists, interferon receptor agonists, and IL-12 agonists; (4) Immunosuppression and Regulation: Strategies focused on controlling excessive immune activation through immunosuppressive agents; (5)Other Specific Mechanisms: Interventions with clearly defined biological roles not covered by the above categories, such as DNA inhibitors, telomerase inhibitors, and IDO inhibitors.

### 2.5 Data analysis and quality control

The analysis was primarily descriptive. Categorical variables (e.g., trial phase, vaccine platform, endpoints) were summarized using frequencies and percentages. Temporal trends were analyzed by plotting the annual number of trial initiations. To ensure data accuracy and reliability, a dual independent review process was implemented (20). Two researchers independently extracted data from the Trialtrove database, and the resulting datasets were cross-validated. Any discrepancies were resolved through discussion or, when necessary, arbitration by a senior researcher. The inter-rater agreement prior to consensus, calculated using the kappa statistic, was excellent (κ > 0.85).

## 3 Results

### 3.1 Conflict between geographical concentration and disease heterogeneity: misalignment of R&D landscape with global disease burden

**Figure 1:**
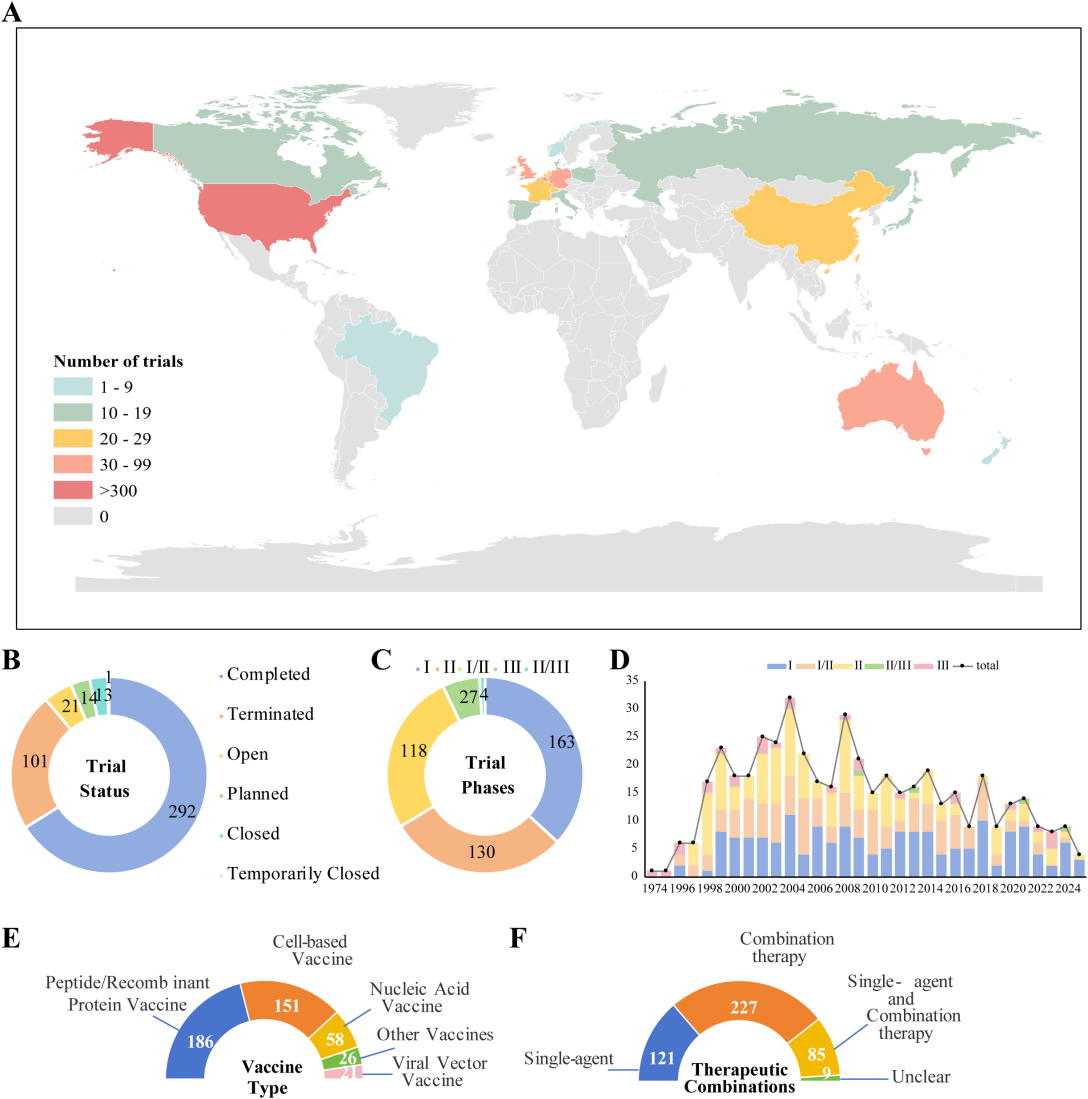
**(A)** Global distribution of melanoma vaccine clinical trials; **(B)** Status of melanoma vaccine clinical trials; **(C)** Phase distribution of melanoma vaccine clinical trials; **(D)** Annual initiation of melanoma vaccine trials by hhase; **(E)** Distribution of melanoma vaccine trials by delivery platform; **(F)** Therapeutic strategies in melanoma vaccine trials.

Data revealed a significant misalignment between the global distribution of melanoma vaccine R&D resources and the geographical characteristics of disease burden. In terms of regional distribution, 69.9% of trials (n=310) were concentrated in North America, and 28.5% (n=126) in Western Europe (**Figure 1A**). In contrast, regions with a high prevalence of non-cutaneous melanoma subtypes—such as acral melanoma (predominant in Asia) and mucosal melanoma (relatively high in Asia and Africa)—had trial participation rates of less than 5%. A more critical conflict lies in the “subtype-technology mismatch” caused by geographical imbalance: analysis of antigen targets across 442 trials showed that over 95% of R&D designs were entirely based on cutaneous melanoma (the dominant subtype in Caucasian populations), with core targets focusing on driver gene mutations such as BRAF and NRAS. In stark contrast, only 5 trials were designed for acral melanoma-specific vaccines—this subtype is characterized by molecular features including telomerase reverse transcriptase (TERT) promoter mutations and KIT abnormalities, which are fundamentally distinct from the driver gene profile of cutaneous melanoma. This mismatch directly creates a clinical applicability gap: even if existing vaccines complete Phase III validation and gain market approval in Europe and North America, their benefits cannot cover nearly 40% of global patients with non-cutaneous melanoma. Consequently, the geographical concentration of R&D resources is not merely an issue of resource allocation, but has become the primary barrier restricting the “global accessibility” of melanoma vaccines.

### 3.2 Structural barriers to clinical translation: the translational funnel and imbalanced R&D funding system

**Figure 2:**
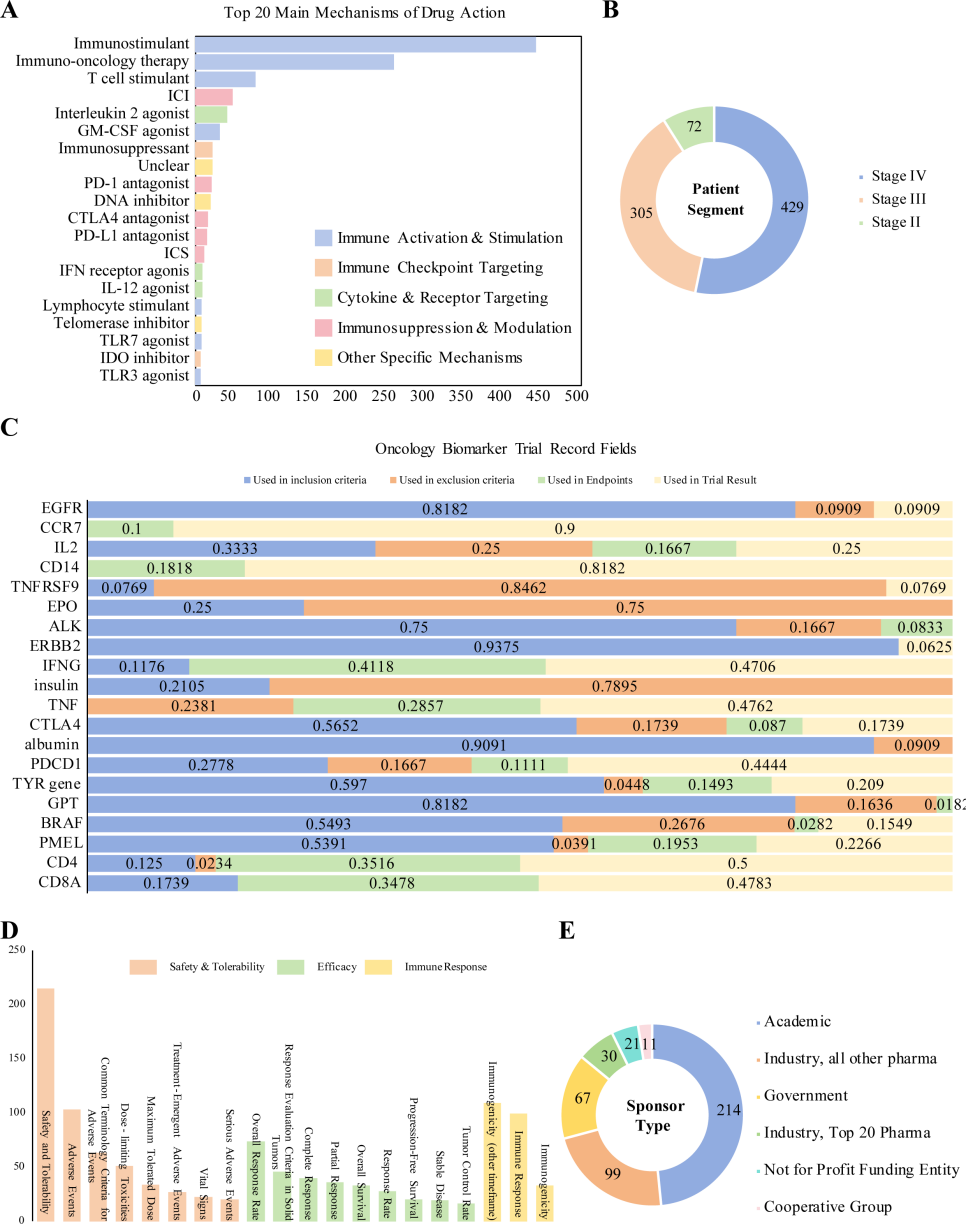
**(A)** Top mechanisms of action in melanoma vaccine trials; **(B)** Distribution of melanoma vaccine trials by disease stage; **(C)** Distribution of oncology biomarkers across clinical trial phases; This figure illustrates the proportional utilization of various oncology biomarkers in different aspects of clinical trials, including inclusion criteria, exclusion criteria, endpoint assessment, and result analysis. Values represent the proportion of trials in which each biomarker was used for the specified purpose. Blue: Trial inclusion phase. Orange: Trial exclusion phase. Green: Endpoint assessment phase. Yellow: Results analysis phase. **(D)** Primary endpoints in melanoma vaccine trials; **(E)** Distribution of melanoma vaccine trials by sponsor type.

Analysis of the trial phase distribution reveals a significant "translational funnel" dilemma in melanoma vaccine R&D: early-phase trials (Phase I/I-II) constitute a high proportion of 63.6% (n=281), whereas only 6.1% (n=27) progress to Phase III for final efficacy validation. The overall termination rate across all phases reaches 22.9% (n=101), indicating inefficient transition from early exploration to late-stage development (**Figures 1B, C**). Structural bias in clinical endpoint design further exacerbates this challenge. Safety/tolerability (215 trials) and immunogenicity-related endpoints (142 immunogenicity trials, 99 immune response trials) dominate the landscape. In contrast, "hard endpoints" directly linked to clinical benefit, such as Overall Survival (OS, 33 trials) and Progression-Free Survival (PFS, 20 trials), are utilized in a very low proportion of trials (**Figure 2D**). A typical manifestation is observed with some peptide vaccines, which can induce robust T-cell activation in Phase I. However, these T cells often exhibit a PD-1⁺TIM-3⁺ terminally exhausted phenotype and lack the ability to effectively infiltrate the tumor microenvironment (TME), highlighting the disconnect between early immune markers and clinical benefit. Imbalance in R&D entities and funding structure constitutes a core driver of the translational obstacle. Academic institutions lead 52.3% of trials (n=231), with their strengths concentrated in early proof-of-concept and immunogenicity exploration. However, they face significant limitations in the substantial funding required for Phase III trials, multi-center management, and industrial translation capabilities. Industry participation remains low at only 31.9% (n=141), resulting in a fractured pipeline from "early exploration – late-stage validation – industrialization" (**Figure 2E**). Furthermore, funding from government and non-profit organizations accounts for only 21.7% (n=96), which is insufficient to cover the high costs of Phase III trials. The application of precision detection technologies, such as multimodal biomarker assessment and single-cell RNA sequencing, can increase costs for single-center early-phase trials by 50%–80%. Constrained by funding, the utilization rate of such technologies is generally low in academically-led trials, further restricting the quality improvement of early-stage R&D.In summary, current melanoma vaccine R&D faces multiple structural contradictions: the coexistence of redundant early-phase trials and inefficient late-stage translation, overreliance on immunogenicity surrogate endpoints with insufficient linkage to clinical benefit, and an imbalance between academic leadership in early R&D and a lack of industry-provided resources for late-stage development. Coupled with stage mismatches in funding allocation and the cost pressures of applying advanced technologies, these issues collectively intensify the clinical translation bottleneck, presenting a core challenge for the field's advancement.

### 3.3 Iterative dilemma of technical platforms: “involution” of mature pathways *vs*. “breakthrough” potential of emerging platforms

From a temporal perspective, melanoma vaccine R&D can be divided into three phases:1974–1995 (Early Exploration Phase): The core goal was to verify the technical feasibility of “vaccine-induced anti-tumor immunity,” with an average of fewer than 3 trials initiated annually. 1996–2008 (Rapid Expansion Phase): Driven by the rise of tumor immunotherapy, the average annual growth rate reached approximately 13.19%, marking the entry into a large-scale exploration period. Post-2009 (Platform Integration and Transformation Phase): The number of annually initiated trials stabilized (peaking at 38 in 2018), and fluctuations in trial numbers over the past five years indicate that the field has entered a new stage driven by “technical platform iteration” (**Figure 1D**). In terms of technical platform distribution, mature platforms dominated the field: peptide/recombinant protein vaccines (186 trials, 42.1%) and cellular vaccines (151 trials, 34.1%) accounted for a combined 76.2% (**Figure 1E**). However, the developmental bottlenecks of these two platforms have become increasingly prominent: Peptide vaccines: Despite their widespread application, they rely on adjuvants (e.g., interleukin-2 (IL-2) agonists: 42 trial; granulocyte-macrophage colony-stimulating factor (GM-CSF) agonists: 32 trials) to overcome their inherent limitation of weak immunogenicity (**Figure 2A**). Cellular vaccines (e.g., dendritic cell (DC) vaccines): Although they possess potent antigen-presenting capacity, they are constrained by “autologous cell sources, complex preparation processes, and long production cycles,” making large-scale clinical application difficult. In contrast, nucleic acid vaccines (particularly mRNA platforms), despite accounting for only 13.1% of trials (n=58), have demonstrated disruptive potential. The landmark trial NCT03897881 (mRNA-4157 combined with pembrolizumab) showed that this regimen reduced the risk of recurrence or death by 49% in high-risk postoperative melanoma patients. Its advantages stem not only from efficacy but also from its technical characteristics: it can rapidly encode multiple neoantigens (e.g., covering 34 neoantigens in a single dose) to address tumor heterogeneity, and its industrialized production process resolves the “accessibility” challenge faced by cellular vaccines—providing a key direction for platform iteration.

### 3.4 Divergence in combination strategies: homogenization tendency *vs*. precision-based case

Combination therapy has become the mainstream strategy in melanoma vaccine R&D, accounting for 51.4% of trials (n=227) (**Figure 1F**). From a mechanism-oriented perspective, most trials exhibited homogenization characteristics: they focused on combinations of “immune stimulation + immune checkpoint inhibition” (e.g., T-cell stimulation combined with PD-1/PD-L1 inhibitors). Among these, 78 trials adopted T-cell stimulation mechanisms, and 49 trials combined immune checkpoint inhibitors (**Figure 2A**)—all with the core goal of “overcoming TME immunosuppression,” but lacking precise matching to patient subgroups. A small number of precision-based combination cases have verified the value of “mechanism-patient characteristic matching”: NCT03744238 (oncolytic viral vaccine RP1 combined with nivolumab): For patients with immune checkpoint inhibitor (ICI) resistance, viral lysis of tumor cells remodeled the “cold TME” and reversed T-cell exhaustion, ultimately achieving an objective response rate (ORR) of 58.3%. This case indicate that the core value of combination therapy lies not in “combination itself,” but in customizing regimens based on patients’ clinical scenarios (e.g., ICI resistance, postoperative MRD) and biological characteristics (e.g., TME status).

### 3.5 Missed population intervention windows and biomarker systems: emphasis on enrichment over prediction

Data showed that 91.1% of trials (n=403) focused on advanced-stage (Stage III/IV) patients (**Figure 2B**), significantly missing the optimal window for vaccine intervention. Stage II patients have a postoperative recurrence risk of 30%, yet the degree of TME immunosuppression in these patients is far milder than that in advanced-stage patients (the proportion of exhausted T cells is only 1/3 of that in Stage IV patients), making it easier to induce effective immune responses. Results from NCT02890330 confirmed the value of “early intervention”: this trial used TLPLDC vaccine adjuvant therapy for Stage II patients, with 39% of patients achieving clinical benefits and 46% remaining disease-free at a median follow-up of 22.5 months. This suggests that shifting the R&D focus “forward” to the Stage II adjuvant therapy phase is a critical strategic direction to improve the success rate of melanoma vaccine R&D. In terms of biomarkers, prospective patient enrichment strategies based on statically enriched driver genes (e.g., ERBB2/HER2 (93.8%), EGFR (81.8%), BRAF (54.9%)) have been widely applied to screen patient subgroups with matched targets. In contrast, dynamic predictive biomarkers that reflect efficacy (e.g., CD8A (34.8% for endpoint application/47.8% for result analysis), IFNG (41.2%/47.1%), CD4 (35.2%/50.0%)) (**Figure 2C**), as well as novel biomarkers (e.g., circulating tumor DNA (ctDNA) clearance rate, T cell receptor (TCR) clonal diversity), remain largely limited to retrospective analysis. Only a small number of trials have incorporated them into prospective predictive designs. This lag in the “prediction-evaluation” system further exacerbates the high failure rate of Phase III trials and has become another key barrier to clinical translation.

## 4 Discussion

Our analysis of 442 clinical trials reveals a complex developmental landscape in this field characterized by "coexisting technical potential and translational dilemmas": On one hand, the R&D of vaccines targeting cutaneous melanoma—including those combining BRAF/NRAS inhibitors with antigen-based vaccines—has advanced with robust evidence. Two Phase III clinical trials (median follow-up > 5 years) showed that, versus placebo, this combination regimen significantly reduced the risk of recurrence or death by 48% (hazard ratio [HR] for recurrence-free survival [RFS] = 0.52) and the risk of distant metastasis or death by 44% (HR for distant metastasis-free survival [DMFS] = 0.56)(21); On the other hand, challenges persist, including the regional monopolization of R&D resources, the "Caucasian population bias" in clinical trials (which disconnects early-stage R&D from the global disease spectrum), the lag in research on the molecular mechanisms of subtypes, insufficient exploration of subtype-specific antigens for non-cutaneous melanoma, and inefficient vaccine delivery—a critical yet underintegrated barrier (22).

To address the bottleneck of geographical bias and "Caucasian population bias," there is an urgent need to establish a "disease spectrum-oriented" global collaborative R&D system, with the following key measures: First, establish intercontinental R&D consortia (e.g., the "Asia-Africa Melanoma Vaccine Clinical Trial Network") coordinated by the World Health Organization (WHO), integrating resources from academic institutions, pharmaceutical companies, and regulatory authorities across countries. Funding sources should be diversified, including WHO special funds, joint investments from multinational pharmaceutical companies, and matching government funds (e.g., China’s Belt and Road Health Cooperation Fund)(23, 24). Simultaneously, establish unified data collection standards, mutual recognition of ethical reviews (referencing the Guidelines for Ethical Collaboration in International Multicenter Clinical Trials to realize mutual recognition among China’s NMPA, US FDA, and European EMA), and standardized adverse event reporting processes to shorten trial initiation time. This ensures that populations from high-incidence regions in Asia, Africa, and Latin America are included in Phase I/II trial designs (rather than merely as late-stage validation cohorts), guaranteeing representative population coverage throughout R&D. Second, prioritize funding for the R&D of subtype-specific antigen vaccines by setting up a "Non-Cutaneous Melanoma Vaccine R&D Special Fund" (with contributions from international pharmaceutical companies, charitable organizations such as the Melanoma Research Alliance [MRA], and governments of high-incidence countries in a 1:1:1 ratio) and simplifying application procedures. Provide early R&D grants for projects targeting key mutations (e.g., TERT promoter mutations, KIT abnormalities in acral/mucosal subtypes) and mandate data sharing via the WHO Global Cancer Database Platform to break "data silos" and accelerate target validation(25).Third, promote international regulatory authorities (e.g., FDA, NMPA) to develop "subtype adaptability evaluation criteria" by establishing an interagency working group to formulate specialized efficacy indicators for acral/mucosal subtypes, issuing a "Subtype-Specific Vaccine Data Submission Guide", and adopting a "Real-Time Oncology Review" model to shorten approval cycles. This institutional adjustment will drive the globalization of the R&D landscape, truly realizing the "global accessibility" of melanoma vaccines and ensuring that technological innovations benefit patients with all melanoma subtypes.

Beyond geographical bias, the core root of the "translational funnel" phenomenon in tumor vaccine R&D—characterized by a large number of early-phase trials and a high Phase III attrition rate—lies in the disconnection between early-phase trial design and ultimate clinical success. This disconnect stems from the synergistic effect of multiple factors: systematic bias in clinical endpoint design, a fractured academia-industry translation pipeline, structural imbalances in funding allocation, insufficient linkage between preclinical and clinical research, and insufficient consideration of inefficient delivery barriers. In the "go/no-go" decision-making of early-stage tumor vaccine R&D, while over-reliance on immunogenicity and safety as primary endpoints has limitations, their core practical value has made them standard choices—safety assessment can mitigate R&D risks (e.g., viral vector vaccines for prostate cancer avoided severe adverse events through early safety monitoring) (26), and immunogenicity can quickly complete proof-of-concept (e.g., in vitro detection of T-cell proliferation and IFN-γ release to confirm vaccine activation).

However, this reliance has obvious drawbacks, exacerbated by inefficient delivery: a robust T-cell response observed in Phase I may fail due to T-cell exhaustion or inability to infiltrate the TME (27); more critically, even functionally competent T cells or vaccines themselves often fail to reach tumor sites due to inefficient delivery systems (28), rendering peripheral blood immunogenicity signals clinically meaningless (29). Compounding the problem, the fractured academia-industry pipeline and funding imbalance further amplify the translational dilemma: academic institutions lead 52.3% of trials but lack resources for Phase III studies and industrialization capabilities, while corporate participation is only 31.9%, leading to a broken "early exploration-late validation-industrialization" chain; the massive funding required for Phase III trials (often exceeding $1 billion per project) lacks sustainable supply (only 15% of R&D funding flows into Phase III) (30), and academic institutions’ limited budgets hinder the application of cutting-edge technologies such as multimodal biomarkers and single-cell RNA sequencing, impeding the upgrade of endpoint assessment systems.

From a technological evolution perspective, the tumor vaccine field is undergoing a fundamental paradigm shift, providing critical support for addressing the aforementioned translational bottlenecks. The field has long been committed to improving antigen delivery efficiency and immune response intensity, with technical platforms evolving gradually from traditional peptide vaccines to next-generation systems represented by mRNA. Early short peptide vaccines had low delivery efficiency and easily induced T-cell tolerance (31), while subsequent synthetic long peptides (SLPs) improved cross-presentation efficiency through epitope fusion and modification (32); synergistic innovations in adjuvants and delivery systems have further broken through bottlenecks—novel adjuvants (e.g., the TLR3/MDA5 agonist poly-ICLC, STING agonists) have replaced aluminum adjuvants, and delivery strategies such as lipid nanoparticles (LNPs) and albumin hitchhiking have enhanced lymphatic targeting, resolving the issues of insufficient vaccine uptake and limited activation in previous generations (33, 34). The emergence of the mRNA platform (e.g., mRNA-4157) has ushered in a new stage of multi-targeted, rapid-production vaccine development, overcoming stability and delivery challenges through molecular modification and LNP delivery (33–35), and demonstrating significant advantages in enhancing immune responses and addressing tumor heterogeneity (12). In the future, next-generation platforms integrating biomimetic nanocarriers, intelligent adjuvants, and efficient delivery systems will lay the foundation for truly personalized immunotherapy and potent anti-tumor effects.

Building on technological advancements, combination therapies have become a dominant paradigm in cancer immunotherapy, but their success is not inevitable. While numerous studies have demonstrated that combining cancer vaccines with other immunotherapeutic agents can improve the TME and enhance efficacy, our analysis reveals a concerning trend toward homogenization—predominantly pairing vaccines with immune checkpoint inhibitors (ICIs)(36, 37). Truly compelling efficacy data come from rationally designed combinations tailored to specific clinical contexts: for example, in ICI-resistant patients, oncolytic viruses convert "cold" TME to "hot" TME to enhance vaccine efficacy (37); in melanoma patients, personalized neoantigen peptide vaccines combined with nivolumab induce epitope spreading and prolong progression-free survival; mRNA-4157 combined with pembrolizumab achieved a 96% 2.5-year overall survival rate in high-risk melanoma patients, significantly outperforming monotherapy.

The core mechanisms of these combinations include: vaccine-ICI restoring exhausted T-cell function, vaccine-chemo/radiotherapy modulating the TME through immunogenic cell death, vaccine-CAR-T extending cell persistence, and vaccine-STING agonists enhancing T-cell priming efficiency (38,39). Despite promising outcomes, combination therapies face challenges: STING gene mutations leading to agonist non-responsiveness, risks of systemic inflammatory toxicity, and long production cycles and high costs of personalized vaccines. Future efforts must shift from empirical mixing to mechanism-driven design, through in-depth biomarker analysis and real-time immune monitoring, developing novel STING agonists, optimizing delivery strategies to reduce toxicity, and identifying shared neoantigens for off-the-shelf vaccine development to promote broader application (40, 41).

To comprehensively address these interconnected challenges, a mechanism-driven, multidimensional systematic solution must be established, with key strategies outlined as follows: 1. Upgrade the early endpoint assessment system: Move beyond assessing only immune response intensity to integrate "quality-durability-TME adaptability-delivery efficiency," detecting T-cell cytotoxic function, memory subset retention, TME remodeling, and delivery-related indicators (e.g., vaccine accumulation at tumor sites, lymph node targeting efficiency)(42); prioritize cost-effective core biomarker panels (e.g., peripheral blood antigen-specific T-cell frequency + ctDNA dynamic monitoring) and promote multi-center shared testing platforms to reduce costs (42, 43). 2. Optimize the preclinical-to-early clinical transition: Use patient-derived organoid models to screen vaccine candidates capable of inducing effective T-cell killing and compatible with efficient delivery systems (e.g., LNPs, biomimetic nanocarriers): integrate immune, molecular, clinical, and delivery-associated biomarkers to precisely enrich responsive patient populations (44). 3. Drive the transformation of clinical trial design: Advance the intervention window to postoperative minimal residual disease (MRD) or neoadjuvant stages, exploring synergistic regimens of vaccine + ICI + optimized delivery systems; integrate single-cell RNA sequencing and mass cytometry to dynamically monitor immune responses and delivery efficiency, adopt adaptive designs to adjust protocols in real time, and establish a multimodal endpoint framework encompassing "immune-clinical-patient-reported outcomes-delivery parameters" (44, 45). 4. Strengthen academia-industry collaboration and funding systems: Establish early-phase joint R&D mechanisms where enterprises pre-invest in cutting-edge technical detection for academic teams in exchange for priority in late-stage project translation; promote joint funding by governments, non-profits, and enterprises, setting up Phase III special funds and precision testing subsidies, and leveraging regulatory pathways such as FDA Breakthrough Therapy and EMA PRIME to reduce enterprise risks (46, 47). 5. Balance funding distribution across stages: Guide more capital toward late-stage R&D, co-establish "Translational Medicine Platforms" to shepherd early projects through Phase III and industrialization, and set up "Early-Stage Technology Incubation Funds" to support process optimization and cost control, bridging the funding gap between innovation and industrialization. In summary, implementing these interconnected strategies—integrating global collaborative frameworks, technological advancements, precision combination therapies, and robust academia-industry funding mechanisms—will effectively mitigate the risk of disconnection between early R&D and late-stage clinical translation, addressing key barriers such as geographical bias, delivery-immune-endpoint gaps, academia-industry fractures, and suboptimal combination therapy design while accommodating the funding capacity of academic institutions to ensure feasibility. Ultimately, this will drive the transformation of tumor vaccine R&D from an "experience-dependent" paradigm to a "mechanism-driven, precision-oriented" one, laying a critical foundation for its broad clinical application and ensuring that technological innovations benefit patients with all melanoma subtypes worldwide.

## 5 Limitations

This study has several limitations that do not undermine the scientific validity of its core conclusions: First, the analysis relied solely on the Trialtrove database, introducing inherent selection bias that is not fully mitigated by its integrated design—while Trialtrove aggregates data from major registries (e.g., ClinicalTrials.gov, WHO ICTRP) and minimizes duplication/inconsistencies (a strength validated in cancer immunotherapy systematic reviews), its curation standards and update lags may omit regional trials, small-scale academic-led studies, or non-Western regional registries, which could slightly overestimate enterprise-funded trial proportions and underestimate non-Western R&D activity, affecting the granularity of conclusions on geographic distribution and funding structure. Additionally, the study focused on macro-level trial characteristics (e.g., phase, endpoints, funding) and lacked micro-level data, precluding exploration of individualized mechanisms underlying translational bottlenecks, and publication bias cannot be ruled out as negative/inconclusive trials may be underreported. Notably, these limitations primarily impact the generalizability of supplementary conclusions rather than core findings, and future research should address this by conducting cross-database validation and supplementing micro-level data to enhance the depth and generalizability of conclusions.

## 6 Conclusion

Melanoma vaccines represent a promising frontier in cancer immunotherapy, particularly when integrated with immune checkpoint inhibitors and personalized platforms. However, their clinical translation remains hampered by several critical challenges: significant geographical disparities in R&D focus, a high attrition rate in late-phase trials due to endpoint misalignment, technological limitations of traditional platforms, and a narrow focus on advanced-stage patients. To overcome these barriers, a multifaceted strategy is essential. This includes fostering global collaboration to ensure equitable representation of diverse melanoma subtypes, advancing next-generation platforms (e.g., mRNA-based vaccines) for enhanced immunogenicity and scalability, shifting therapeutic windows toward earlier disease stages, and implementing mechanism-driven combination therapies tailored to individual immune contexts. Ultimately, the successful translation of melanoma vaccines will depend on interdisciplinary cooperation, the adoption of predictive biomarker systems, and supportive regulatory frameworks. Only through these concerted efforts can we realize the full potential of melanoma vaccines as a broad-spectrum and globally accessible therapeutic option.

## Data Availability

The datasets presented in this study can be found in online repositories. The names of the repository/repositories and accession number(s) can be found below: https://clinicalintelligence.citeline.com.
