# Supplementary material for: Melanoma vaccines: current R&D landscape, translational hurdles, and future outlook—a perspective drawn from 442 clinical trials
Source: Front Immunol. 2026 Jan 19;16:1698503. doi: 10.3389/fimmu.2025.1698503 (PMC12862056; doi:10.3389/fimmu.2025.1698503)

Supplementary Material

## Supplementary Figures

**Figure 1:**

A:Global Distribution of Melanoma Vaccine Clinical Trials;

B:Status of Melanoma Vaccine Clinical Trials;

C: Phase Distribution of Melanoma Vaccine Clinical Trials;

D:Annual Initiation of Melanoma Vaccine Trials by Phase;

E:Distribution of Melanoma Vaccine Trials by Delivery Platform

F:Therapeutic Strategies in Melanoma Vaccine Trials

**Figure 2：**

A:Top Mechanisms of Action in Melanoma Vaccine Trials;

B: Distribution of Melanoma Vaccine Trials by Disease Stage;

C: **Distribution of Oncology Biomarkers Across Clinical Trial Phases；**

**This figure illustrates the proportional utilization of various oncology biomarkers in different aspects of clinical trials, including inclusion criteria, exclusion criteria, endpoint assessment, and result analysis. Values represent the proportion of trials in which each biomarker was used for the specified purpose.**

**Blue: Trial Inclusion Phase**

**Orange: Trial Exclusion Phase**

**Green: Endpoint Assessment Phase**

**Yellow: Results Analysis Phase.**

D:Primary Endpoints in Melanoma Vaccine Trials;

**E:Distribution of Melanoma Vaccine Trials by Sponsor Type**

Figure1


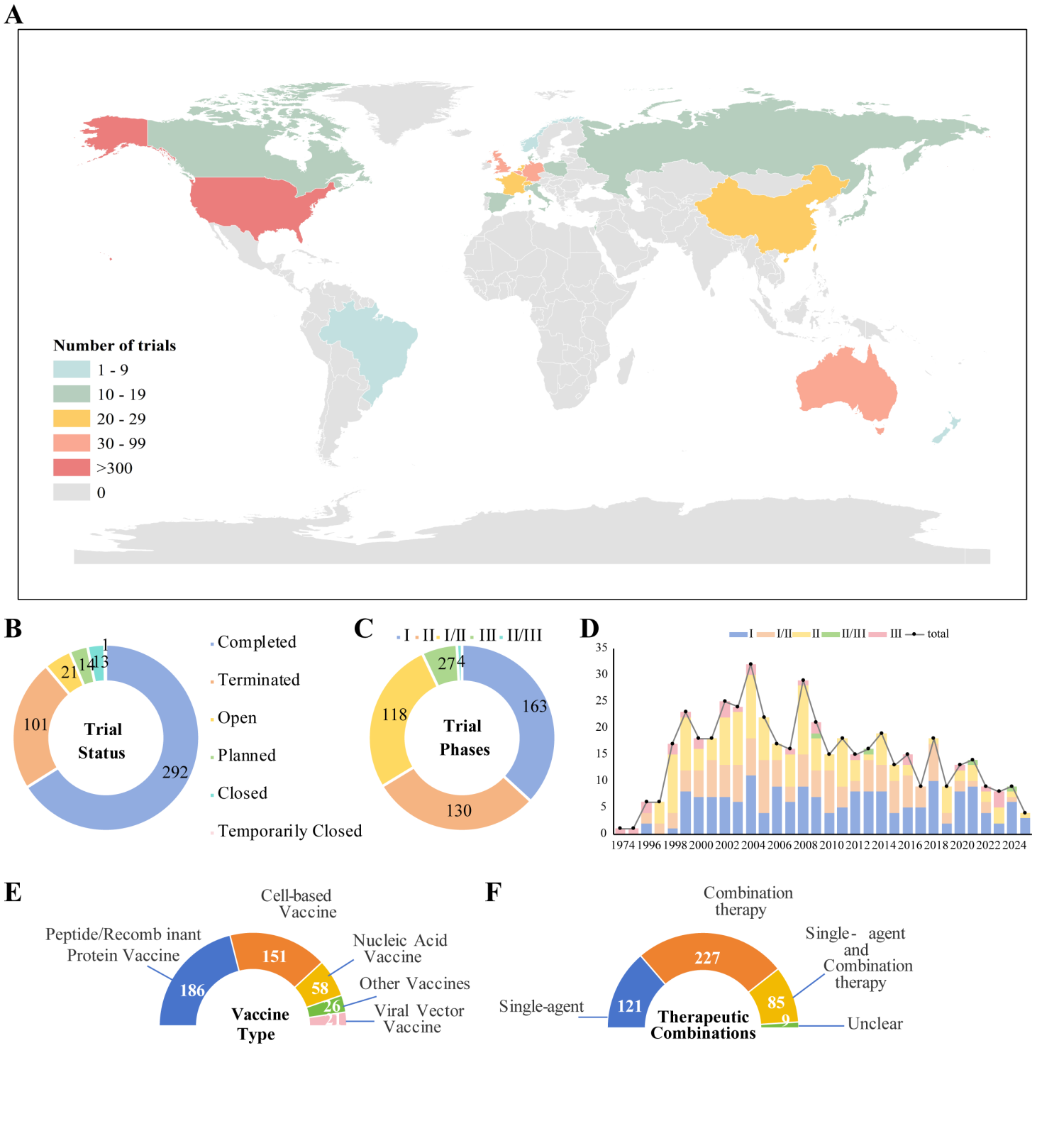


Figure2


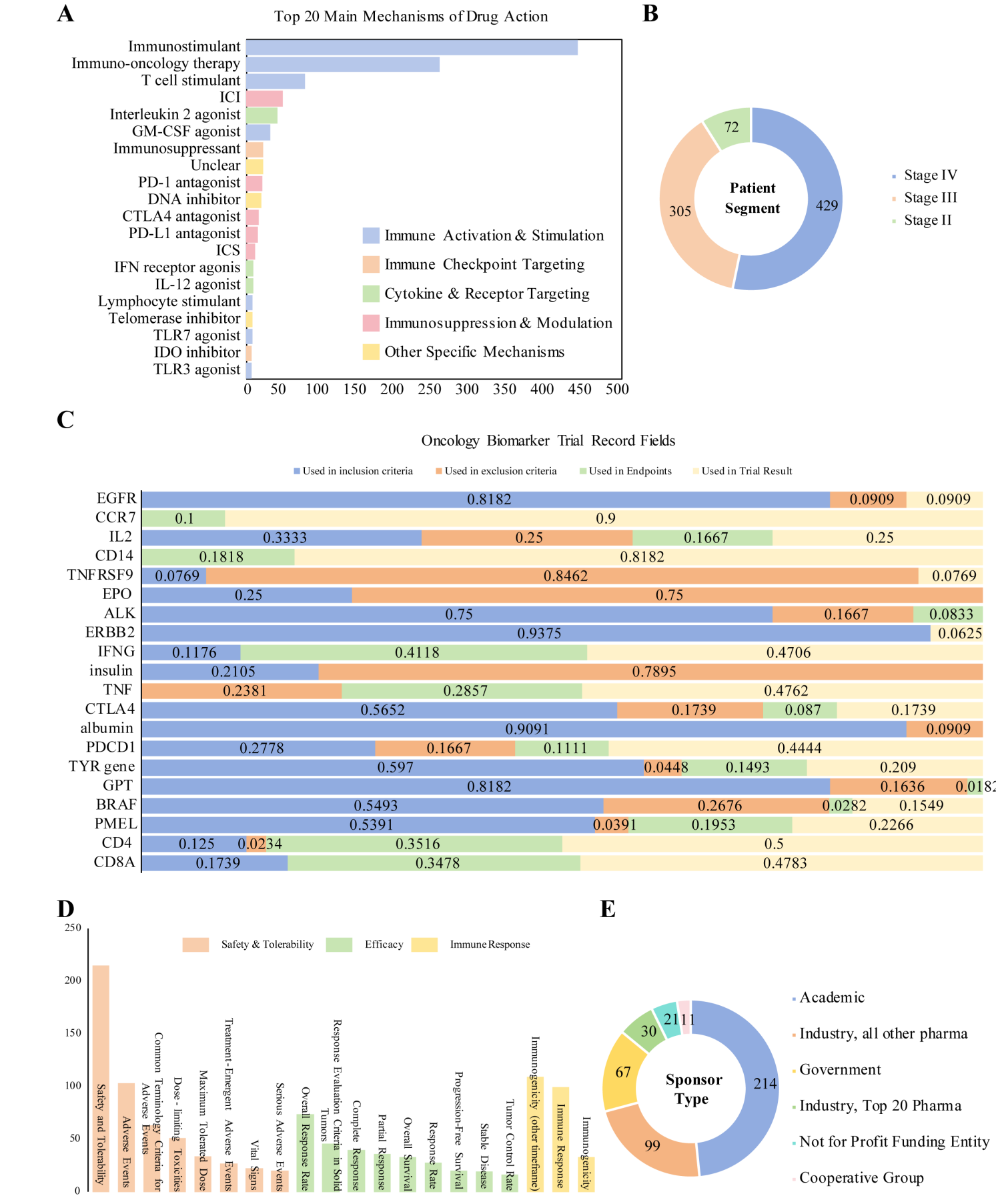

Supplement: Supplementary file 1 [file Table1.docx]
